# Monocytes as Endothelial Progenitor Cells (EPCs), Another Brick in the Wall to Disentangle Tumor Angiogenesis

**DOI:** 10.3390/cells9010107

**Published:** 2020-01-01

**Authors:** Filipa Lopes-Coelho, Fernanda Silva, Sofia Gouveia-Fernandes, Carmo Martins, Nuno Lopes, Germana Domingues, Catarina Brito, António M Almeida, Sofia A Pereira, Jacinta Serpa

**Affiliations:** 1 CEDOC, Chronic Diseases Research Centre, NOVA Medical School|Faculdade de Ciências Médicas, Universidade NOVA de Lisboa, Campo dos Mártires da Pátria, 130, 1169-056 Lisboa, Portugal; filipa.coelho@nms.unl.pt (F.L.-C.); fernanda.silva@nms.unl.pt (F.S.); sofia.gopf@live.com.pt (S.G.-F.); germana.andreia@gmail.com (G.D.); sofia.pereira@nms.unl.pt (S.A.P.); 2 Instituto Português de Oncologia de Lisboa Francisco Gentil (IPOLFG), Rua Prof. Lima Basto 1099-023 Lisboa, Portugal; mcmartins@ipolisboa.min-saude.pt (C.M.); amalmeida1@gmail.com (A.M.A.); 3 Instituto de Biologia Experimental e Tecnológica, Avenida da República, Estação Agronómica, 2780-157 Oeiras, Portugal; nlopes@ibet.pt (N.L.); anabrito@ibet.pt (C.B.); 4 Instituto de Tecnologia Química e Biológica António Xavier, Universidade Nova de Lisboa, Av. da República, 2780-157 Oeiras, Portugal; 5 Hospital da Luz, Av. Lusíada 100, 1500-650 Lisboa, Portugal

**Keywords:** monocytes, endothelial progenitor cells (EPCs), endothelial cells (ECs), angiogenesis, cancer

## Abstract

Bone marrow contains endothelial progenitor cells (EPCs) that, upon pro-angiogenic stimuli, migrate and differentiate into endothelial cells (ECs) and contribute to re-endothelialization and neo-vascularization. There are currently no reliable markers to characterize EPCs, leading to their inaccurate identification. In the past, we showed that, in a panel of tumors, some cells on the vessel wall co-expressed CD14 (monocytic marker) and CD31 (EC marker), indicating a putative differentiation route of monocytes into ECs. Herein, we disclosed monocytes as potential EPCs, using in vitro and in vivo models, and also addressed the cancer context. Monocytes acquired the capacity to express ECs markers and were able to be incorporated into blood vessels, contributing to cancer progression, by being incorporated in tumor neo-vasculature. Reactive oxygen species (ROS) push monocytes to EC differentiation, and this phenotype is reverted by cysteine (a scavenger and precursor of glutathione), which indicates that angiogenesis is controlled by the interplay between the oxidative stress and the scavenging capacity of the tumor microenvironment.

## 1. Introduction

Tumor angiogenesis is essential to provide oxygen and nutrients to cancer cells, and to remove waste products from the tumor microenvironment. Therefore, neovascularization has a crucial role during carcinogenesis, by promoting tumor growth and invasion and ultimately leading to metastasis [1,2]. Over the last two decades, cancer treatment using anti-angiogenic therapies has fallen short of expectations [3], showing that the mechanisms underlying neovascularization in cancer are not fully understood. This failure can be explained, at least in part, by the fact that the specific origin of endothelial progenitor cells (EPCs) is not yet determined.

Over the last decade, different studies reported EPCs as essential in restoring injured vessels. EPCs belong to a subset of cells, arising from hematopoietic stem cells in bone marrow; upon pro-angiogenic stimuli, they proliferate, migrate, and differentiate into endothelial cells (ECs) [4,5,6]. Some reports addressing EPCs and disease, such as systemic sclerosis, showed contradictory and discrepant results about EPCs mobilization and differentiation; in part, because there is a lack of a precise panel of cell surface markers used for the characterization of this subset of cells [4,5,6,7,8,9,10]. In mouse embryonic vascular endothelium, erythro-myeloid progenitors (EMPs) can differentiate into ECs [11] and in a mouse model of carotid injury, monocytes (CD14^+^ cells) are capable of improving re-endothelialization [12]. In vivo and in vitro targeting of Tie2-monocytes decreases angiogenesis by abrogating EC proliferation [13,14,15] and an in vivo CCR2 (chemokine (C–C motif) receptor 2) knockout impairs monocytes recruitment and VEGFA (also named VEGF, vascular endothelial growth factor) expression, accompanied by a reduction in the angiogenesis rate [16]. The release of cytokines and pro-angiogenic factors (e.g., VEGFA, VEGFC, and VEGFD, TNFα (tumor necrosis factor α), IL-8 (interleukin-8), and FGF-2 (fibroblast growth factor-2), and extracellular matrix (ECM) modifying proteins (e.g., metalloproteinase-9 (MMP-9)) by macrophages enhances the tissue’s ability to support capillary sprouting and vascular density [17,18]. The precise mechanism by which monocytes influence angiogenesis in tissue development, homeostasis, and diseases is not fully understood. However, different studies, have shown that under in vitro pro-angiogenic pressure, blood mononuclear cells can acquire endothelial markers and morphology [19,20,21]. In addition, in a previous study, we showed that some ECs simultaneously expressed CD14 (monocytic marker) and CD31 (EC marker), indicating mixed features between monocytes and ECs, in tumors and normal tissues [22]. We truly believe that monocytes are promising candidates for EPCs.

Reactive oxygen species (ROS) are reported as essential for the maintenance of endothelium homeostasis by acting as physiological regulators of intracellular signaling, whilst a redox imbalance can prompt vascular diseases [23,24,25,26,27,28]. Nevertheless, some contradiction persists amongst biological models. In a rat model, hydrogen peroxide (H_2_O_2_) inhibits the differentiation of rat multipotent adult progenitor cells (MAPCs), including bone marrow stem cells and EPCs [29,30,31], whereas it enhances the rate of cell differentiation of human embryonic stem cells [32]. Based on these contradictory observations, we believe the effects of ROS will depend on the cell lineage and its differentiation level.

Herein, we propose to prove, in vitro and in vivo, that monocytes can act as EPCs, being able to incorporate in the neo-vasculature and favor cancer development. Moreover, we used H_2_O_2_ (ROS) as a stimulus to trigger monocyte–EC differentiation.

## 2. Materials and Methods

### 2.1. Monocytes Isolation and Culture

Monocytes were isolated from peripheral blood, collected under consenting donation of healthy donors from *Serviço de Imuno-Hemoterapia* at *Instituto Português de Oncologia de Lisboa Francisco Gentil* (IPOLFG) (IPOLFG-Ethical committee UIC-1137). Peripheral blood mononuclear cells (PBMCs) from blood samples were separated using Histopaque-1077 (10771, Sigma-Aldrich, Saint Louis, MO, USA), followed by magnetic monocytes isolation using a Monocyte isolation kit II (130-091-153, MACS Technology-MiltenyiBiotec, Bergisch Gladbach, Germany), according to the manufacturers’ protocols. Monocytes were cultured in plates coated with 0.2% gelatine (G-1890, Sigma-Aldrich) or with Matrigel (354230, Corning, NY, USA) and maintained in Colony-Forming Unit (CFU) medium (130-091-277, MACS Technology-MiltenyiBiotec) or Endothelial-Basal Medium-2(EBM-2) (CC-3156, Lonza, Basel, Swiss) plus Endothelial-cell Growth Medium (EGM^TM^-2) bullet kit SingleQuotsTM Supplements (CC-4176, Lonza) and with 2% fetal bovine serum (FBS; CC4101A, Lonza), 50 ng/mL vascular endothelial growth factor (VEGF; V7259, Sigma-Aldrich) and 10 U/mL heparin (H3149, Sigma-Aldrich). Cells were maintained at 37 °C, in a humidified atmosphere and 0.5% CO_2_. Hydrogen peroxide, (15 µM; H_2_O_2_; 1.07210.0250, Merck, Saint Louis, MO, USA) was used as a ROS generator, cysteine (0.4 mM; Cys; 7048-04-6, Merck) was used as an anti-oxidant, and disulfiram was used as an ALDH (aldehyde dehydrogenase) inhibitor (2 µM; 86720, Fluka, Munich, Germany).

### 2.2. Cell Culture

Human umbilical vein endothelial cells (HUVEC; ATCC^®^ CRL-1730™) were seeded in plates coated with 0.2% gelatine and cultured in EBM-2 (CC-3156, Lonza) plus EGMTM-2 SingleQuotsTM Supplements (CC-4176, Lonza) medium supplemented with 2% FBS.

Breast cancer cells (MDA-MB-231; ATCC^®^ HTB-26™, and HCC1954; ATCC^®^ CRL 2338™) were cultured in DMEM - Dulbecco’s Modified Eagle Medium (DMEM) (11965-092, Gibco-Thermo Fisher Scientific, Waltham, MA, USA) supplemented with 10% FBS and 1% Antibiotic-Antimycotic (15240062, Invitrogen™—Thermo Fisher Scientific) and Roswell Park Memorial Institute (RPMI)- 1640, no phenol red (#11835-063, Invitrogen, Waltham, MA, USA) supplemented with 10% FBS, 1% Penicillin and streptavidin (15140-163, Gibco-Thermo Fisher Scientific), 0.5 mL 2-β-Mercaptoetanol (21985-023, Gibco-Thermo Fisher Scientific) and 3 mL HEPES (4-(2-hydroxyethyl)-1-piperazineethanesulfonic acid; 15630-056, Gibco-Thermo Fisher Scientific) (respectively). Cells were maintained at 37 °C, in a humidified atmosphere and 5% CO_2_.

### 2.3. Cell Characterization by Flow Cytometry

Adherent monocytes-derived cells were detached with 2 mM EthylenedDiamine TetraAcetic acid-Phosphate Buffer Saline (EDTA-PBS) (*v*/*v*) and incubated with anti-CD14 (1:100; 555397, BD Bioscience, Franklin Lakes, NJ, USA), anti-CD31 (1:100; FAB3567A, R&D Systems, Abingdon, UK), anti-VE- (Vascular-Endothelial Cadherin) (1:100; FAB1002A, R&D Systems), anti-KDR (Kinase insert Domain Receptor) (1:100; FAB357A, R&D Systems), anti-CD68 (1:100; 333810, Biolegend, San Diego, CA, USA), anti-CD80 (1:100; 305206, BioLegend), and anti-CD163 (1:100; 333626, Biolegend). Briefly, cells were incubated with antibodies in 0.5% PBS-Bovine Serum Albumin (BSA) (A9647, Sigma-Aldrich) (*v*/*w*), for 20 min, in dark at 4 °C. Von Willebrand factor (vWF; or factor VIII) detection was performed with anti-vWF (1:500; A0082, Dako—Agilent, Santa Clara, CA, USA), for 60 min at 4 °C with gentle shaking. After incubation, cells were rinsed and re-incubated with a secondary antibody (Alexa Fluor^®^ 488 anti-rabbit-A-11078, Invitrogen-Thermo Fisher Scientific), for 30 min at 4 °C and in the dark with gentle shaking. The immunolabelling was evaluated by flow cytometry (FACScalibur–Becton Dickinson, Franklin Lakes, NJ, USA) and data were analysed using FlowJo (http://www.flowjo.com/) software (Becton Dickinson).

### 2.4. Reverse Transcription and Quantifying PCR (RT-qPCR)

Total RNA was extracted using a RNeasy Mini Extraction kit (74,104; Qiagen, Hilden, Germany) and cDNA was synthesized using SuperScript II Reverse Transcriptase (18080e44, Invitrogen-Thermo Fisher Scientific), according to the manufacturer’s protocol. Relative quantification using qPCR was performed using Power SYBR Green PCR Master Mix (4367659, Applied Biosystems-Thermo Fisher Scientific), according to manufacturer’s instructions, and carried out in a LightCycler 480 instrument (Roche, Basel, Swiss). The primers used are presented in Table 1.

### 2.5. 3D Co-Culture and Microencapsulation

For the co-cultures, HCC1954 cells were inoculated at a density of 0.2 × 10^6^ cell/mL in a 125 mL impeller spinner vessel (Corning) and aggregated at 80 rpm for 4 days. The spheroids were then co-cultured with human derived monocytes and encapsulated in 1.1% (*w*/*v*) Ultra-Pure Ca^2+^ medium viscosity high-guluronic acid (MVG) alginate (UP MVG NovaMatrix, Pronova Biomedical, Avaldsnes, Norway), prepared in NaCl 0.9% (*w*/*v*). The alginate microencapsulation was performed on a VARV1 encapsulator (Nisco, Zurich, Switzerland) to obtain capsules of approximately 700 µm and the polymerization occurred in a 20 mM BaCl_2_ solution. After polymerization, the capsules were washed in NaCl 0.9% (*w*/*v*) and cultured in RPMI 1640, in 125 mL vented cap Erlenmeyer flasks (Corning) at 100 rpm, in orbital agitation. After culture, samples were collected and fixed in 4% (*w*/*v*) formaldehyde with 4% (*w*/*v*) sucrose in PBS for 20 min. Then, they were dehydrated in 30% (*w*/*v*) sucrose for approximately 5 h and embedded in Tissue-Tek^®^ O.C.T. (Sakura, Saint Torrance, CA, USA) and frozen at −80 °C for cryosectioning. The frozen samples were sliced with a thickness of 10 µm in a cryostat (Cryostat CM 3050 S, Leica, Wetzlar, Germany).

### 2.6. Murine (BALB-c\SCID) Model of Neo-Angiogenesis

The animal handling and experimental procedures were performed under the rules of Federation for Laboratory Animal Science Associations (FELASA), accomplishing the 3Rs through evidence-based guidelines. Ethical committee NOVA Medical School (Ref. 75/2019/CEFCM) Female BALB-c/SCID mice (8 weeks) were maintained in a pathogen-free barrier room in the Animal Care Facility at Chronic Diseases Research Center (CEDOC)|NOVA-Medical School (NMS).

In the plug assay, monocytes isolated from the peripheral blood of healthy donors (1 × 10^6^ cells/mice) were previously cultured for 24 h in EBM-2 supplemented with 50 ng/mL VEGF plus 10 U/mL heparin. After, cells were embedded in 400 µL of matrigel supplemented with 50 ng/mL VEGF and subcutaneously inoculated in the right flank of the mice for 21 days. Control groups were inoculated with matrigel supplemented with VEGF. Plugs were removed by surgery and fixed with 2% paraformaldehyde for 10 min, followed by successive 15 min incubations in 100%, 95%, and 60% ethanol and then processed into paraffin-embedded blocks.

Breast tumors were induced by inoculation of MDA-MB-231 cells in the mammary fat pad. Female mice were injected with 50 µL of matrigel with breast cancer cells (MDA-MB-231, 1 × 10^6^ cells/mice) alone and in combination with monocytes, which were previously cultured for 24 h in EBM-2 supplemented with 50 ng/mL VEGF (MDA-MB-231, 1 × 10^6^ cells/mice plus monocytes 1 × 10^4^ cells/mice). Tumors were removed by surgery 45 days after inoculation and fixed with formaldehyde with further inclusion in paraffin-embedded blocks. The tumor volume was calculated using the formula: tumor volume = (length × width^2^)/2.

### 2.7. Immunofluorescence

In vitro cells were cultured on glass slides with 0.2% gelatin coating and fixed in 2% PFA, for 15 min at 4 °C. After blocking, cells were incubated with primary antibodies diluted in 0.1% BSA-PBS (*v*/*w*), overnight (1:100-CD14, ab181470, Abcam, Cambridge, UK; 1:50 -CD31, SCR023, Merck Millipore, Burlington, mA, USA; 1:500-vWF, SCR023, Merck Millipore; 1:500-CD146, SCR023, Merck Millipore), followed by an incubation with secondary antibody for 2 h (1:1000 0.1% BSA-PBS (*v*/*w*), Alexa Fluor^®^ 594 goat anti-rabbit, A-11037, Invitrogen-Thermo Fisher Scientific; Alexa Fluor^®^ 488 goat anti-rabbit, A-11078, Invitrogen-Thermo Fisher Scientific; Alexa Fluor^®^ 488 goat anti-mouse, 115-545-003, Invitrogen-Thermo Fisher Scientific), at room temperature.

Paraffin sections (2 μm) were deparaffinized in xylol for 30 min and re-hydrated in decreased concentrations of ethanol (95%, 70%, and 30%), followed by an immersion in water and antigen recovery using citrate buffer. Slides were blocked with 5% BSA-PBS (*w*/*v*) for 30 min to avoid non-specific interactions, followed by an incubation with 0.3 M glycine-goat serum-0.1% Triton ×100, for 30 min. After, slides were incubated with primary antibodies diluted in 1% BSA-PBS (*v*/*w*) (vWF-1:200, A0082; Dako; FN-1:50, SAB4500974, Sigma-Aldrich), for 1 h at room temperature followed by an incubation with secondary antibody (1:500 in 1% BSA-PBS (*v*/*w*); Alexa Fluor^®^ 488 goat anti-rabbit; Alexa Fluor^®^ 594 goat anti-rabbit-A-11037, Invitrogen-Thermo Fisher Scientific), for 2 h, at room temperature. Slides were mounted in VECTASHIELD media with DAPI (4′-6-diamidino-2-phenylindole; Vector Labs) and examined by standard fluorescence microscopy using an Axio Imager.Z1 microscope (Zeiss, Oberkochen, Germany). Images were acquired and processed with CytoVision^®^ software (Leica).

### 2.8. Murine (BALB-c\SCID) Aorta Ex Vivo Model of Vascular Repair

Endothelial injury was induced in murine aortas, longitudinally open, through the exposure to Lipopolyssacharides (LPS) (0.5 µg/mL; L2880-Sigma-Aldrich) plus Lysophosphatidic acid (LPA) (0.5 µg/mL; L7260-Sigma-Aldrich), over 24 h. Afterwards, aortas were rinsed with 1× PBS and embedded in Matrigel with 50 ng/mL VEGF plus 10 U/mL heparin with human Day 3 monocytes, cultured in EBM2 supplemented with 50 ng/mL VEGF plus 10 U/mL heparin, for 5 days. Aortas were removed from Matrigel and fixed with 4% paraformaldehyde (PFA) for 2 h, and rinsed with blocking buffer (1× PBS + 10% horse serum + 0.3% Triton X-100). The aortas were incubated with primary antibody diluted in 1% BSA-PBS (*v*/*w*) (vWF—1:200, A0082; Dako), overnight at 4 °C, followed by two times rinsing with blocking buffer and a 2 h incubation with secondary antibody (1:500 in 1% BSA-PBS (*v*/*w*); Alexa Fluor^®^ 594 goat anti-rabbit) at room temperature. After the final washes with blocking buffer, aortas were mounted in slides with in VECTASHIELD media with DAPI. Images were acquired using an LSM 710 (Zeiss) confocal microscope equipped with a Plan-Apochromat 63/1.40 Oil Ph3 lens and Zen Blue 2010b SP1software (2010b; Zeiss).

### 2.9. Fluorescence In Situ Hybridization (FISH)

After immunofluorescence analysis, FISH using an alphoid probe for centromere X (pLAX) [33], and a probe for the Yq12 region (pHY2.1) [34] was applied to the same slides/tissue sections having anti-human vWF positive cells. The X-centromere probe was labeled with digoxigenin (DIG) and the Yq12 probe with biotin, as previously described [35]. Briefly, slides were incubated twice in 2xSSC for 5 min at room temperature to wash away the DAPI antifade solution, re-hydrated in ethanol series, and incubated in citrate buffer, in a water bath for 10 min at 90 °C. Enzymatic digestion was carried out with 4 mg/mL pepsin in 0.2N HCl (pH 2.0) for 30 min at 37 °C. After that, 10 μL diluted probes were co-denatured with the tissues at 90 °C for 5 min and hybridized at 37 °C, overnight. Post-hybridization wash was performed in 2× SSC/0.3% NP-40 for 3 min at 73 °C followed by a wash in 2× SSC/0.1% NP-40 for 2 min at room temperature. X-centromere DIG-labeled probe was detected by anti-digoxigenin-FITC (Fluorescein isothiocyanate) (Anti-digoxigenin- fluorescein, Fab fragments; 11270741910, Roche), biotinylated Yq12 probe was detected by Cy3-streptavidin (CyTM3-conjugated Streptavidin; 016-160-084, Jacksons Lab, Bar Harbor, ME, USA), and nuclei were counterstained with DAPI-vectashield mounting solution. FISH signals, in previously recorded immunofluorescence vWF and FN positive cells, were captured and analyzed using a Zeiss Epifluorescence microscope linked to a Cytovision FISH Probe software program (Cytovision version 7.4, Leica Biosystems, Richmond, VA, USA).

### 2.10. Immunohistochemistry (IHC)

Paraffin sections (2 μm) from paraffin-embedded tissue blocks were deparaffinized, re-hydrated, as mentioned above, and antigen recovery was performed using citrate buffer. Slides were rinsed with 1× Tris Buffered Saline (TBS) followed by the blockage of endogenous peroxidase (K4011, Dako) for 10 min. Anti-vWF (1:200 in IHC diluent) was incubated for 3 h and anti-fibronectin (FN; 1:50 in IHC diluent, RE7133-CE, Leica) for 1h, at room temperature. Staining was performed using ABC detection kit (ab93677, Merck Millipore), according to the manufacturer’s protocol. For nuclear staining, slides were immersed in Mayer’s Hematoxylin (RE7164, Leica) for 5 min. Slides were dehydrated and mounted with Entellan (1079610500, Merck) and digitalized using Aperio ImageScope-Pathology Slide Viewing software version 12.4 (Leica, Wetzlar, Germany).

### 2.11. Data Statistical Analysis

Data were analyzed using t-test and one-way ANOVA tests in GraphPad Prism 7 software (7, Prism GraphPad, San diego, CA, USA). Statistically significant changes were determined at the *p* value ≤ 0.05.

## 3. Results

### 3.1. In Vitro Monocytes Differentiate into Endothelial Cells (ECs)

**Figure 1:**
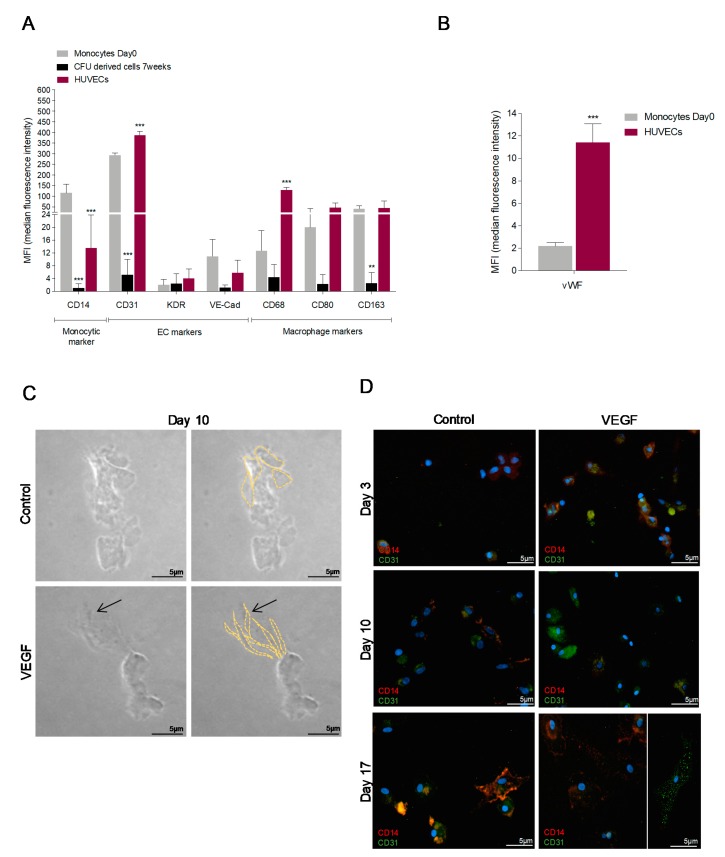

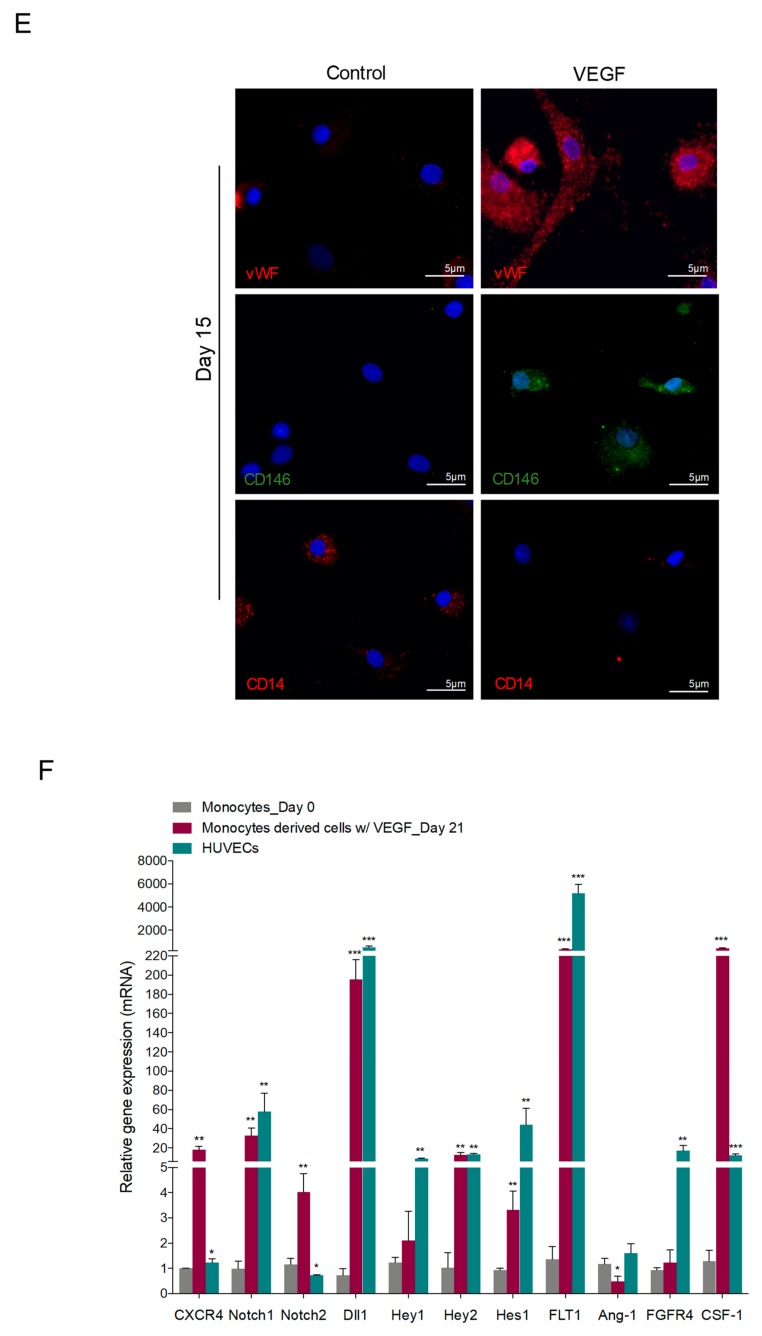
Cultured monocytes undergo an increase in the expression of endothelial cells (ECs) markers and acquire spindle cell like morphology, indicating EC differentiation of monocytes. (**A**) MIF (median intensity fluorescence) values from Flow cytometry analysis of CD14—monocytic marker, CD31, KDR, VE- Cadherin (VE-Cad)—EC markers and CD68, CD80, and CD163—macrophage markers in monocytes freshly isolated (Day 0), monocytes maintained in CFU media and in human umbilical vein ECs (HUVECs). (**B**) MIF (median intensity fluorescence) values from FACS analysis of vWF—EC marker in monocytes freshly isolated (Day 0), and in human umbilical vein ECs (HUVECs). (**C**) Monocytes cultured for 10 days in Matrigel in EBM-2 medium with or without VEGF. Images taken under optical microscopy, magnification 200×; arrow shows spindle shape cells (bars 5 µm). (**D**) Immunofluorescence for CD14 (red) and CD31 (green) in monocytes cultured in EBM-2 medium with or without VEGF for 3, 10 and 17 days (bars 5 µm). DAPI (blue) stained nuclei, magnification 400×. (**E**) Immunofluorescence for CD14 (red) and CD31 (green) in monocytes cultured in EBM-2 medium with or without VEGF for 3, 10 and 17 days (bars 5 µm). DAPI (blue) stained nuclei, magnification 400×. (**F**) Relative quantification of typical endothelial genes in monocytes freshly isolated (Day 0), in monocytes-derived cells cultivated for 21 days in the presence of VEGF and in HUVECs. * *p* ≤ 0.05 ** *p* ≤ 0.01 *** *p* ≤ 0.001.

Freshly isolated monocytes from healthy donors and HUVECs showed a similar profile of endothelial and macrophage markers, with the exceptions of CD14 and vWF that were more expressed, respectively, in monocytes and in HUVECs (Figure 1A,B and Figure S1), pointing out that monocytes cultured in a pro-endothelial medium share molecular features with ECs. Notably, monocytes cultured in CFU media, a media for the maintenance of stem and progenitor cells, had lower expression of endothelial and macrophage markers (Figure 1A), indicating the maintenance of a resting and more undifferentiated state.

Monocytes cultured in matrigel plus VEGF presented a spindle-cell-like morphology (Figure 1C), typical of tip ECs. In the line with the morphological changes observed in cultured monocytes, a dynamic CD31 gain and CD14 loss was observed during 17 days in culture, in the presence of VEGF (Figure 1D). The increase of endothelial markers vWF and CD146 with a concomitant decrease of monocytic marker CD14, were also observed at 15 days of culture, under VEGF stimuli (Figure 1E). In addition, VEGF drives an increase in the expression of endothelium-expressed genes (Notch1, Delta 1 (Dll1), Hes Family BHLH Transcription Factor 1 (Hes1), Hairy/enhancer-of-split related with YRPW motif protein 2 (Hey2), Fms Related Tyrosine Kinase 1 (FLT1), and Colony-Stimulating Factor (CSF)-1) (Figure 1F), which is in accordance with the morphological changes observed in monocytes-derived cells (Figure 1C,D).

### 3.2. Reactive Oxygen Species (ROS) Promote Monocytes Differentiation into Endothelial Cells (ECs)

**Figure 2:**
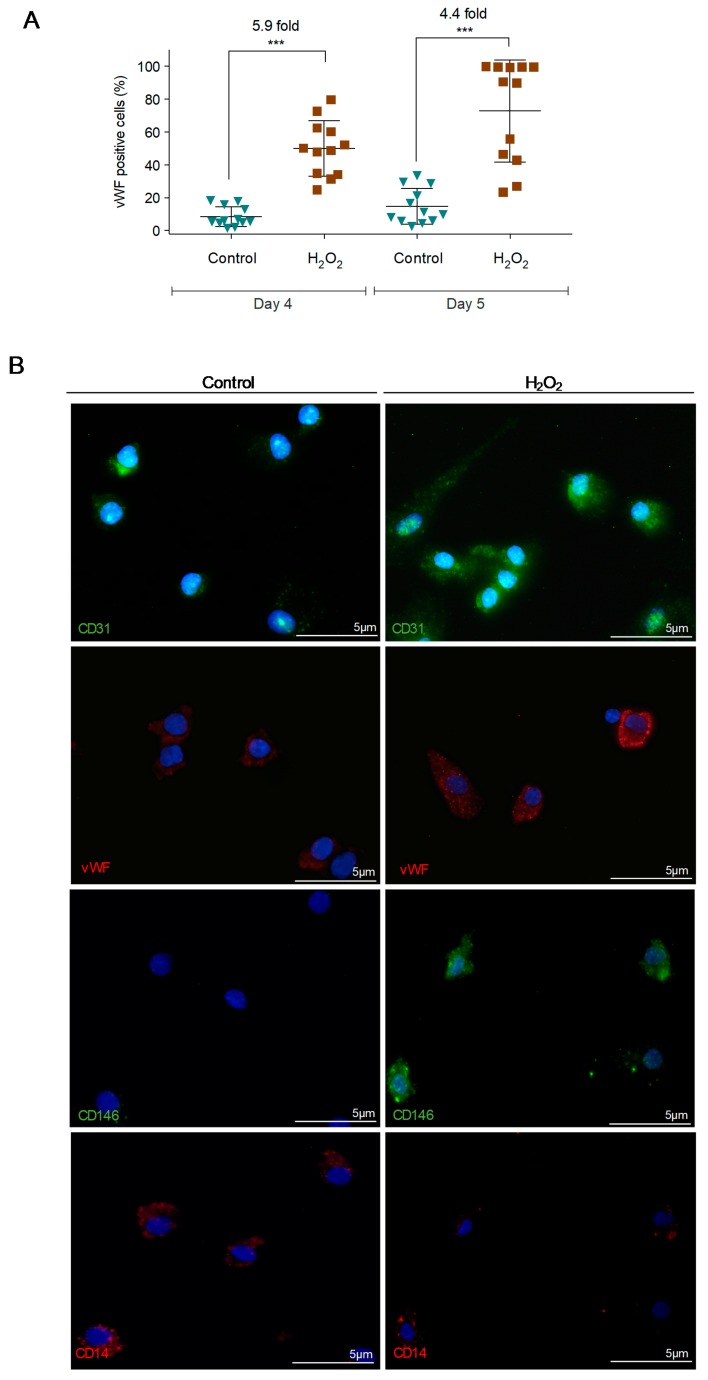

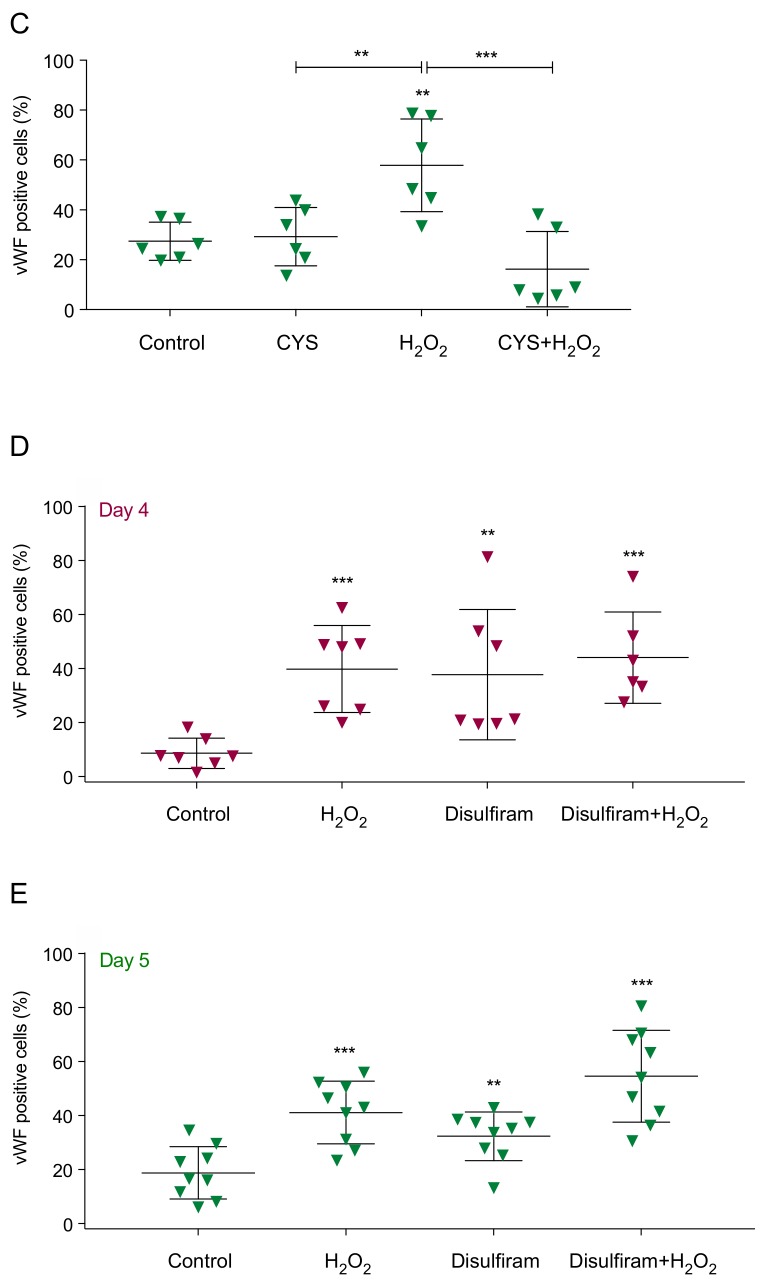
Monocyte-derived cells cultured in the presence of hydrogen peroxide (H_2_O_2_) increase the expression of endothelial cell (EC) markers that is abolished by the presence of cysteine (Cys). (**A**) vWF levels in monocytes-derived cells maintained in CFU media (Day 4) plus 1 day in EBM-2 plus VEGF (Day 5), in the presence or absence of H_2_O_2_ (15 µM). (**B**) Immunofluorescence for CD31 (green), vWF (red), CD146 (green) and CD14 (red) in monocytes cultured during 5 days in EBM-2 medium with VEGF, in the presence or absence of H_2_O_2_ (15 µM). Nuclei are in blue (DAPI), magnification 400× (bars 5 µm). (**C**) vWF levels in monocytes-derived cells maintained in EBM-2 plus VEGF, in the presence and/or absence of Cys (0.4 mM) and H_2_O_2_ (15 µM) for 1 day. (**D**/**E**) vWF levels in monocytes-derived cells maintained in CFU media (Day 4) plus 1 day in EBM-2 plus VEGF (Day 5), in the presence or absence of H_2_O_2_ (15 µM) and/or disulfiram (2 µM). Each dot represents a healthy donor. ** *p* ≤ 0.01 *** *p* ≤ 0.001.

Monocyte-derived cells were exposed to H_2_O_2_ in short term, for 30 min, and showed EC differentiation. This fact was observed in monocytes previously maintained in CFU for 4 days (Day 4) and in monocytes maintained in CFU for 4 days plus 1 day in EBM-2 media (Day 5). The increase in von Willebrand factor (vWF) expression was 5.9- and 4.4-fold, respectively (Figure 2A). The expression of other EC markers, such as CD31 and CD146, was also increased, and CD14 expression was decreased after H_2_O_2_ stimulus (Figure 2B). Cysteine, which is a thiol, precursor of glutathione, and itself an antioxidant, abrogates the H_2_O_2_-induced differentiation process, with no increase in vWF expression (Figure 2C).

Aldehyde dehydrogenase (ALDH) is used as a marker for populations enriched in stem and progenitor cells and a decrease in ALDH expression and activity is correlated with a differentiation status of EPCs to ECs [36,37]. Hence, the impact of ALDH inhibition by disulfiram [38] was evaluated in the monocyte-endothelial differentiation. Monocytes from Day 4 (Figure 2D) and Day 5 (Figure 2E) exposed to disulfiram showed a gain of vWF expression similar to monocytes exposed to H_2_O_2_, reinforcing that monocytes are differentiating into ECs.

### 3.3. In Vivo, Monocytes Differentiate into ECs and Are Incorporated into Blood Vessels

**Figure 3:**
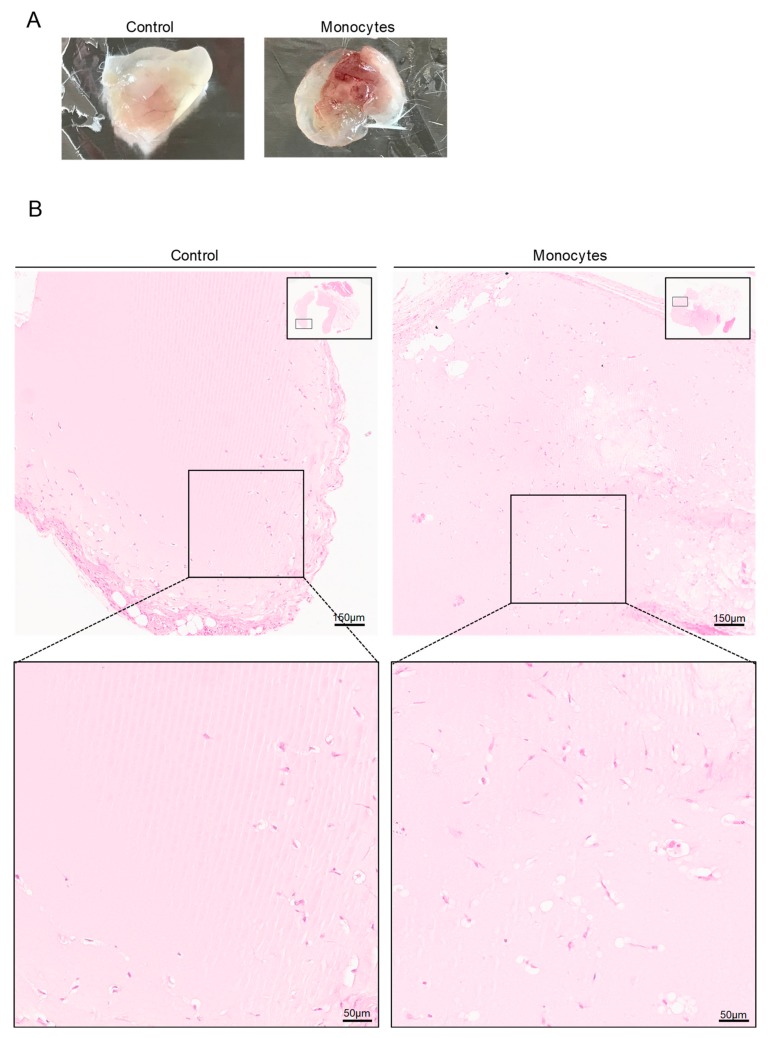

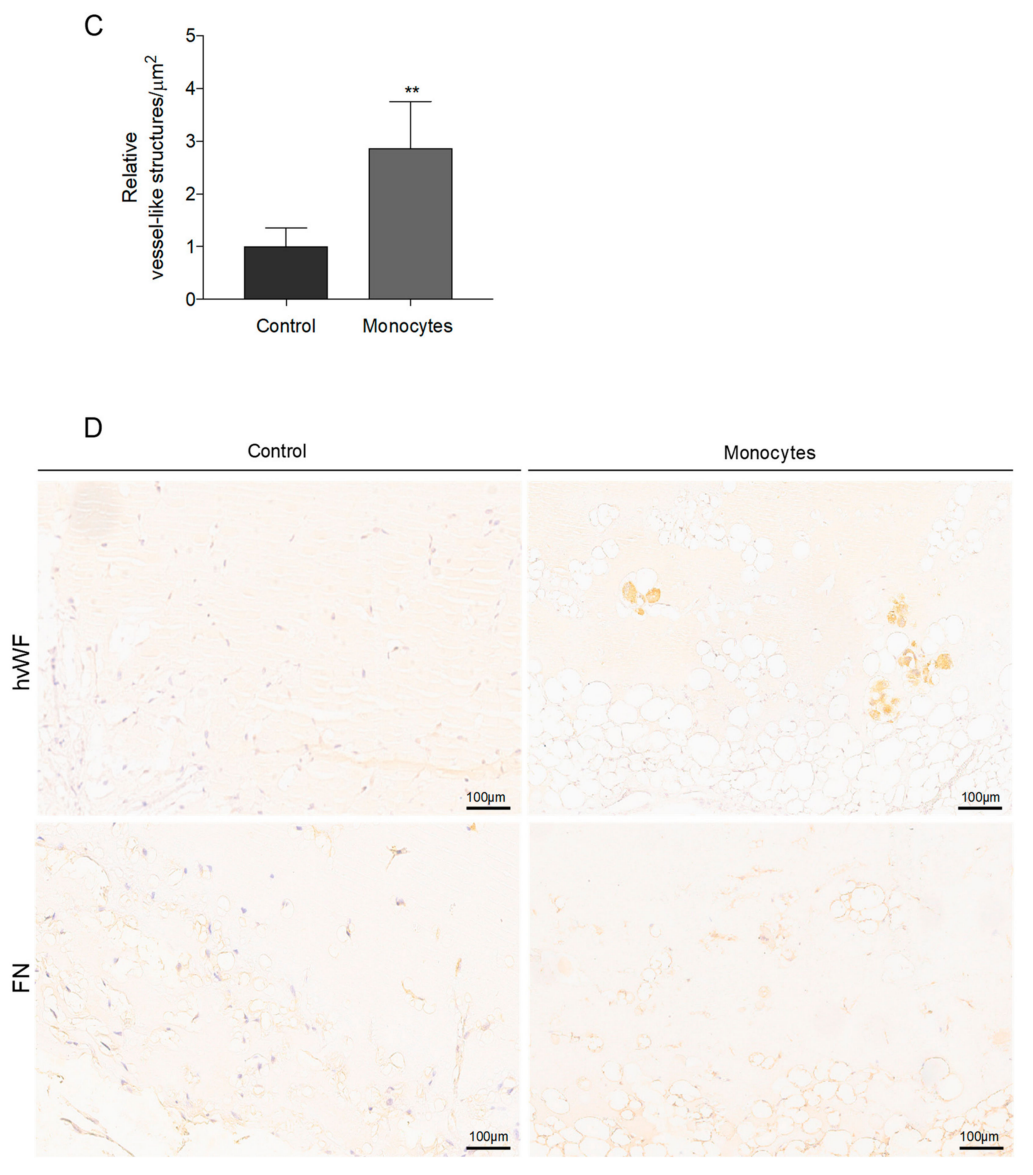

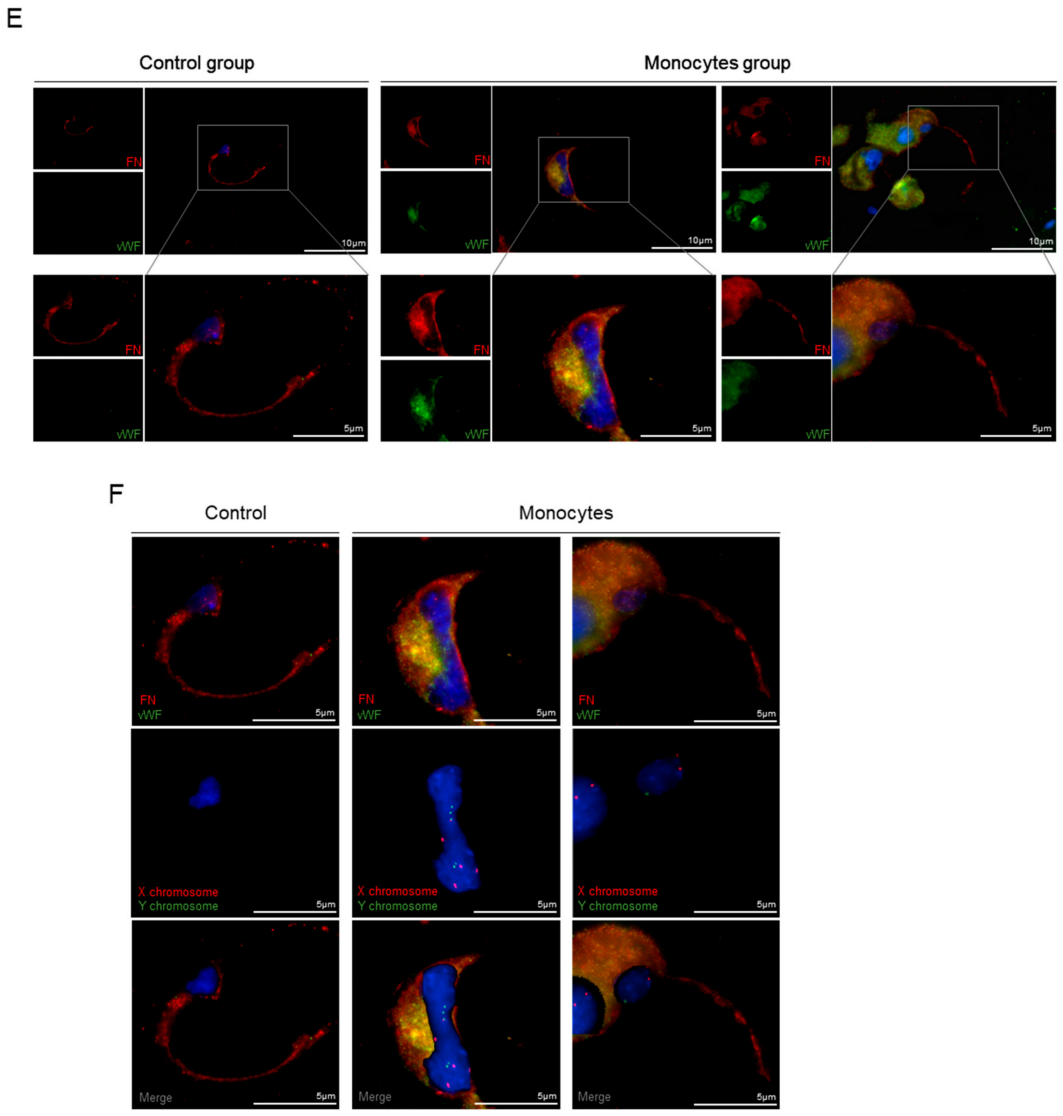
Monocytes exposed to VEGF are able to form blood vessels in vivo. (**A**) Plug images 21 days after monocyte inoculation in matrigel with VEGF. Control plugs were inoculated in the absence of monocytes. (**B**) Hematoxylin and eosin staining from the paraffin embedded plugs (bars 150 µm and 50 µm). (**C**) Relative density of vessel-like structures per area (µm) in plugs (*n* = 4 per group). (**D**) Human CD31 (hCD31) by immunohistochemistry. Optical microscopy, nuclei are blue (hematoxylin) (bars 100 µm). (**E**) Human vWF (hvWF; green) and fibronectin (FN, red) staining by immunofluorescence in plug blood vessels (bars 10 µm and 5 µm). (**F**) The same sections (**E**) were submitted to FISH analysis for the human X (red) and Y (green) chromosome (bars 5 µm). Nuclei are blue (DAPI). ** *p* ≤ 0.01.

Matrigel plugs with embedded human monocytes from male healthy donors were subcutaneously inserted in mice; control plugs were induced with matrigel solely. Macroscopically, plugs with human monocytes exhibited a more exuberant vasculature (Figure 3A) and, microscopically, an increased density of vessel-like structures was observed (Figure 3B,C). Some of the vessel-like structures in plugs containing monocytes were positive for the human vWF (hvWF) (Figure 3D,E and Figure S2), indicating that human monocytes differentiate into ECs and were incorporated into blood vessels. It is noteworthy that the vessel-like structures in control plugs (Figure 3A–C), were negative for hvWF (Figure 3D,E), which confirmed their murine origin. Additionally, vessel-like structures of the control and monocytes group were positive for fibronectin (FN) (Figure 3D,E), a protein of the vessels basement membrane that is expressed by active ECs [39,40], indicating that those structures are new-formed vessels.

In addition to the hvWF expression in monocytes-derived ECs in vessel-like structures, the human and male origin was also confirmed by FISH (Figure 3F) performed with human specific X and Y probes. Thus, we confirmed that the hvWF positive cells, in plugs with monocytes group, were from human origin (X and Y positive; Figure 3F), proving that monocytes can directly differentiate into ECs.

### 3.4. Monocytes Are Able to Repair Injured Aortas

**Figure 4:**
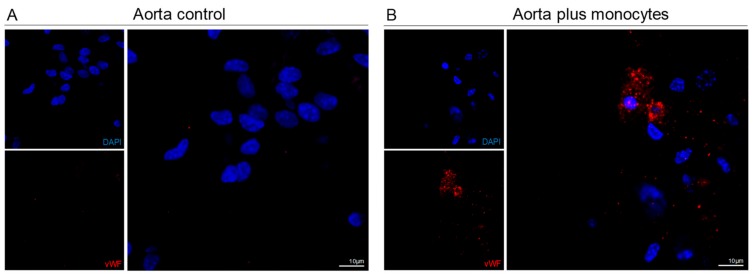
Monocytes are able to be incorporate in the aorta ex vivo, after endothelial Lipopolyssacharides/Lysophosphatidic acid (LPS/LPA)-related injury. Aortas were ex vivo exposed to LPS (0.5 µg/mL) plus LPA (0.5 µg/mL) to induce endothelial dysfunction. Afterwards LPS plus LPA were removed and monocytes were incubated with aortas. (**A**) Murine aorta section incubated with LPS plus LPA, showing no positive cells for human vWF (bars 10 µm). (**B**) Murine aorta section incubated with LPS plus LPA followed by incubation with monocytes, showing positive cells for human vWF (bars 10 µm). Nuclei are blue (DAPI).

In order to verify if monocytes were able to repair injured vessels, we used an ex vivo model of murine aorta injury, due to the exposure to LPA and LPS, which are considered inducer of endothelial dysfunction and injury [41,42,43]. Interestingly, we observed that monocytes incorporated damaged aortas and differentiated into endothelial cells, as revealed by the expression of human vWF (Figure 4).

### 3.5. Monocytes Are Active Stakeholders in Neo-Vasculature Network Formation during Tumor Development

**Figure 5:**
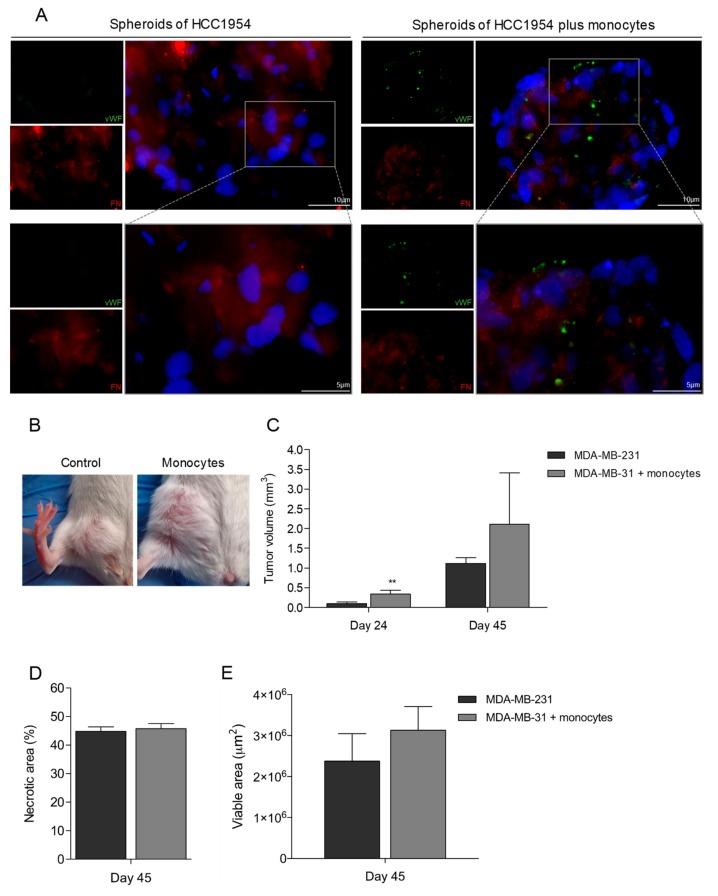

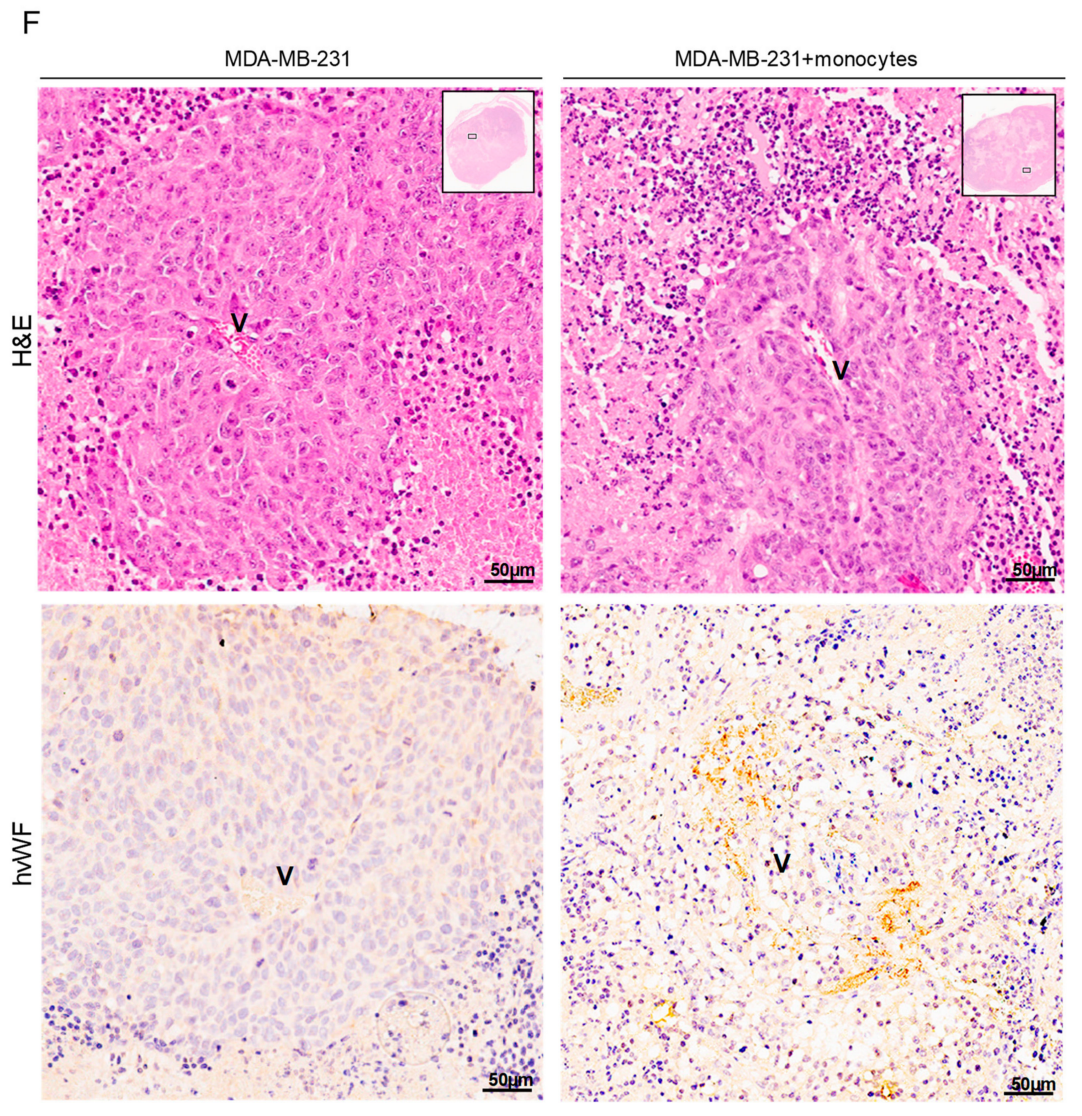
Tumors grown in the presence of monocytes have increased tumor volume and some vessels with human vWF (hvWF) staining. (**A**) 3D spheroids of HCC1954 in co-culture with monocytes stained for hvWF (green) and fibronectin (FN) (red) (bars 10 µm). DAPI (blue) stains nuclei, magnification 400×. (**B**) Representative imagens of tumors from mice with breast cancer cells (MDA-MB-231), without and with monocytes previously cultured under VEGF. (**C**) Tumor volume (mm^3^) 24 and 45 after inoculated mice with breast cancer cells (MDA-MB-231), without and with monocytes previously cultured under VEGF. (**D**) Quantification of tumor necrotic areas, 45 days after the inoculation of MDA-MB-231 with and without monocytes cultured in VEGF. (**E**) Quantification of tumor viable areas, 45 days after the inoculation of MDA-MB-231 with and without monocytes cultured in VEGF. (**F**) Immunohistochemistry (IHC) for hvWF (black arrow) in MDA-MB-231 tumors in the presence and absence of monocytes cultured in VEGF (bars 50 µm). Optical microscopy, nuclei are blue (hematoxylin). Blood vessels are signed with “V”.

To unravel the role of monocytes as EPCs in cancer context, we employed a 3D co-culture system [44] of human breast cancer cells (HCC1954) and human monocytes. In co-culture, hvWF positive cells also expressed FN (Figure 5A), which suggested that monocytes are differentiating into ECs. In control cultures (monocultures of HCC1954), FN labeling was detected but not vWF.

Also in vivo, we investigated if monocytes would be incorporated in the tumoral neo-vasculature, enhancing tumor development and growth. For that, we established a cancer model: Human breast cancer cells (MDA-MB-231) were inoculated in the mammary fat pad of female mice, in the presence or in the absence of human male monocytes. Mice injected with MDA-MB-231 and human monocytes developed larger tumors (4- and 2-fold at days 24 and 45, respectively; Figure 5B,C) in comparison to the mice inoculated only with MDA-MB-231. The percentage of necrotic area was similar in both experimental groups (Figure 5D); the total viable area was more extensive in tumors induced with MDA-MB-231 cells plus monocytes than in tumors induced only by MDA-MB-231 cells (Figure 5E). Remarkably, some ECs in tumor blood vessels of mice inoculated with MDA-MB-231 and monocytes were hvWF positive (Figure 5F; black arrow); in the cancer scenario, that indicates monocytes were able to differentiate into ECs and be incorporated into the neovasculature, contributing to tumor growth.

## 4. Discussion

In addition to their relevance in sprouting angiogenesis, EPC circulation, mobilization, and differentiation also play an essential role in the repair of injured vessels [4,5,6]. In recent years, the role of EPCs in the renewal of ECs integrity and function has gained attention from the scientific community, in particular, from the field of ischemia and cardiovascular disorders, opening new therapeutic perspectives for patient treatments [45,46,47]. So far, the lack of a precise panel of cell surface markers drives some heterogeneity in the methodologies used for EPCs identification. Furthermore, the very low representativeness of EPCs described so far creates a barrier for a better characterization of the molecular and biological processes underlying EPCs function, homing, and differentiation [48,49]. The exact definition of an EPC is still a matter of debate in the scientific community. We wonder if there are other cell subtypes that can also act as EPCs, which are more representative [50] in blood and are not, so far, addressed in studies regarding angiogenesis.

Reports in ischemia disclosed the involvement of bone marrow (BM)-derived mononuclear cells as contributors for angiogenesis, by being incorporated into neocapillaries and by allowing an increased vessel density [51,52,53]. A study from Nolan et al. [54] correlated early stages of tumor development with the differentiation potential of BM-derived EPCs and their incorporation into a subset of tumor neovessels. In a previous study using a panel of normal tissues and of tumors from vascular, epithelial, and embryonic origins, we showed that some cells on the vessel wall co-expressed CD14 (monocytic marker) and CD31 (EC marker) [22]. In accordance to this, another study showed that when macrophages are involved in vascular mimicry or interact with new forming blood vessels, they increase the expression of CD31 [55]. Thus, we hypothesized that monocytes could be involved in neovascularization, by acting as EPCs.

As a matter of fact, in vitro, we verified that freshly isolated monocytes, cultured in an endothelial specific medium (EBM2), exhibited a mixed expression profile of endothelial and macrophages markers (Figure 1A and Figure S1), but expressed very low levels of vWF a bona fide endothelial marker (Figure 1B). Therefore, monocytes gained vWF, together with CD146, expression upon endothelial differentiation (Figure 1E). However, when monocytes were cultured in stem and progenitor cell medium (CFU), they expressed low levels of all those markers (Figure 1A). Monocytes are recognized as a cell type that expresses VEGF receptors (VEGF-R) [56,57] and our results show that, when cultured under the presence of VEFG, monocytes were able to acquire a spindle-cell-like morphology (Figure 1C), typical of ECs. This morphological remodeling was accompanied by an increased expression of CD31, vWF, and CD146 (EC marker) amongst other endothelial specific genes, concomitant with decreased expression of CD14 (monocytic/macrophagic marker) (Figure 1D–F). These in vitro results indicated that monocytes are close to a stem-cell like state, but, upon pro-endothelial/angiogenic stimulation, they differentiate into ECs.

Pathologies like cardiac ischemia or cancer have defective tissue perfusion, and excessive ROS levels and deregulated redox signaling account for re-vascularization of tissues and condition these disease outcomes [58,59,60]. In addition, ROS are essential for endothelium homeostasis and can stimulate or inhibit cell differentiation, depending on the cell type [29,30,31,32,61]. Considering the relevance of EPCs during vascular remodeling in pathologies with alterations in redox balance, we wonder if ROS would impact the monocytic-endothelial differentiation. Under exposure to H_2_O_2_, there was an increase in the expression of specific endothelial markers in monocytes, such as vWF, CD31, and CD146 (Figure 2A) and a decrease in the expression of the monocytic marker CD14 (Figure 2B). It is important to notice that monocytes, both maintained in EBM2 and in CFU media (prior H_2_O_2_ stimulation), were able to react to H_2_O_2_ by increasing the expression of vWF (Figure 2A); this highlights the relevance of ROS in inducing the differentiation of monocyte-derived ECs. The expression of some EC markers, such as CD31, is often observed in macrophagic cells in a cancer context and in vascular mimicry [55,62]. We opt to follow the expression of vWF, since it is in fact a marker for ECs differentiation, as it is expressed and accumulated in Weibel-Palade bodies [63], which are endothelial storage granules. Accordingly, our images showing vWF expression in EC differentiating monocytes demonstrate a granular accumulation, typical of endothelial cells. Moreover, vWF is commonly used in other studies to identify cells undergoing endothelial differentiation [64,65,66].

Recently, in studies dedicated to cancer metabolism, our team showed that cysteine is beneficial for cells coping with ROS [67,68]. In monocytes, cysteine seemed to have the same role, by protecting cells against ROS effects. In this particular case, cysteine stopped the ROS-induced monocyte–ECs differentiation, as it prevented the expression of vWF and impaired the route of monocytes to EC-like differentiation (Figure 2C).

EPCs are characterized by having active ALDH and a decrease in their expression and activity is correlated with a differentiation status of EPCs to ECs [36,37]. In a model of ischemia, decreased expression of ALDH was pointed out as a good strategy to induce rapid neovascularization and subsequent regeneration of ischemic tissues [69]. Our results showed that monocytes exposed to disulfiram, an irreversible inhibitor of ALDH, increased vWF expression in a pattern similar to that observed in monocytes exposed to H_2_O_2_ (Figure 2D,E), this way reinforcing the role of monocytes as EPCs. Thus, the decreased activity of ALDH prompts the monocyte–ECs differentiation process, in a similar manner to ROS (H_2_O_2_). The role of ROS (H_2_O_2_) as a stimulator of monocyte differentiation into ECs was reinforced by the observation that even when monocytes are shortly removed from CFU media, both disulfiram and H_2_O_2_ can induce the expression of vWF (Figure 2D). The evidence is compatible to the high plasticity and capacity of monocytes differentiating into ECs, without requiring a long-term ROS stimulus. The increased ROS levels during disease progression is a feature already assumed in cancer [70]. Therefore, our results reinforce the evidence that the metabolic remodeling of cancer cells contributes to a ROS enriched microenvironment, being a mean of orchestrating tumor neo-angiogenesis and favoring tumor growth and spread [71].

In a tumor microenvironment, stromal cells (e.g., fibroblasts, adipocytes, inflammatory, and smooth muscle cells) and cancer cells secret monocyte chemoattractant protein 1 (MCP-1) that leads to the recruitment of monocytes into the tumor [72,73,74]. Furthermore, the macrophage migration inhibitory factor (MIF), whose function as a regulator of inflammation remains controversial [75,76,77], seems to promote cancer progression by stimulating the recruitment of myeloid cells into the tumor [78,79,80,81], is also associated with increased angiogenesis [82,83]. Since MIF has been directly implicated in the regulation of endothelial differentiation [84], we believe that MIF as a tumor promoter proves again that monocytes-derived ECs can be a subpopulation of M2-TAMs, reaching the tumor to favor angiogenesis by also making part of the blood vessel structures. Interestingly, an oxidized isoform of MIF was identified as a prognostic biomarker and therapeutic target in inflammatory disease and cancer [85,86], showing again that the pro-oxidative tumor microenvironment is playing a role in angiogenesis.

The participation of monocytes in angiogenesis, via the production and release of pro-angiogenic factors, has been reported in inflammatory diseases [87,88,89]. However, our results suggest that monocytes, in addition to their role in the secretion of cytokines, pro-angiogenic factors (e.g., VEGF, VEGFC, and VEGFD, TNFα, IL-8, and FGF-2) and ECM modifying proteins (e.g., MMP-9) [17,18], were also able to integrate new blood vessels by directly differentiating into ECs and incorporating the vessel structure (Figure 3). This phenomenon was observed macroscopically by a higher density of blood vessels in plugs inoculated with monocytes (Figure 3A) with an increased number of vessel-like structures (Figure 3B,C) expressing FN (Figure 3D,E), which indicates that those structures have a vessel basement membrane [90]. Moreover, using two different in vivo models, we unraveled human monocyte capacity to differentiate into ECs and incorporate blood vessels (Figure 3B,C). The strategy we used, by inoculating human male monocytes in plugs induced in female mice, undoubtedly proved that cells positive for hvWF were from human (Figure 3B,C) and male origin (Figure 3F). In addition, it was evident that not all vessel-like structures within the plug were from human origin (Figure 3D), since murine EPCs were also participating in the pro-angiogenic process [91] and might react in a more efficient way as they were reacting to endogenous murine signaling. A recent study, confronting autologous and heterologous EPCs transplantation models stated that in the autologous context, cells undergo homing and differentiation more efficiently [92].

The differentiation of monocytes into endothelial cells, expressing vWF, was also induced in murine aortas ex vivo (Figure 4), after endothelial dysfunction induced by LPS and LPA [41,42,43]. Again, the human origin of those cells incorporating injured aortas was assessed by using a human specific anti-vWF. Several studies have, shown that injured arteries are repaired by both the recruitment of new cells and the activation of the proliferation of endothelial cells [42,93,94,95]. In this experiment, monocytes worked as recruited EPCs, being able to incorporate an already structured but injured vessel.

The efficacy of the use of anti-angiogenic therapies in cancer treatment have been disappointing so far. We believe this failure relies on the missing pieces to construct the entire angiogenic route. Considering that monocytes recruitment is a well-established step during carcinogenesis [96,97], we believe this recruitment favors tumor progression not only by differentiating into tumor associated macrophages (TAMs) [98,99,100,101], but also by acting as EPCs and differentiating into ECs. In a 3D co-culture system of breast cancer cells and monocytes, we detected hvWF positive cells that also expressed FN (Figure 5A). This suggested that, also in in vitro cancer 3D-models, monocytes can differentiate into ECs.

Once in the tumor and upon their activation, some monocytes can differentiate into macrophages that release cytokines and pro-angiogenic factors, whilst other monocytes can be targeted by this stimulation that will trigger their differentiation into ECs. This coordinated myeloid networking will favor tumor growth and disease progression. According to this, we observed, in vivo, that breast cancer cells inoculated in the presence of monocytes originated larger tumors in comparison to the control group (4- and 2-fold at day 24 and 45, respectively; Figure 5B,C). The necrotic area was similar in the two experimental groups (Figure 5D,E), showing that the viable area in tumors induced in the presence of monocytes is more extensive than the viable area in control tumors. This observation can roughly show that monocytes are acting as helpers for tumor development. Interestingly, in the viable regions of tumors inoculated with monocytes, but not in the control group, some ECs and cells close to the vessels were hvWF positive (Figure 5F). Therefore, in both the in vivo murine breast cancer model and the 3D in vitro model, we unraveled that monocytes have the potential to differentiate into ECs and be incorporated into the neo-vasculature, during tumor development.

**Figure 6:**
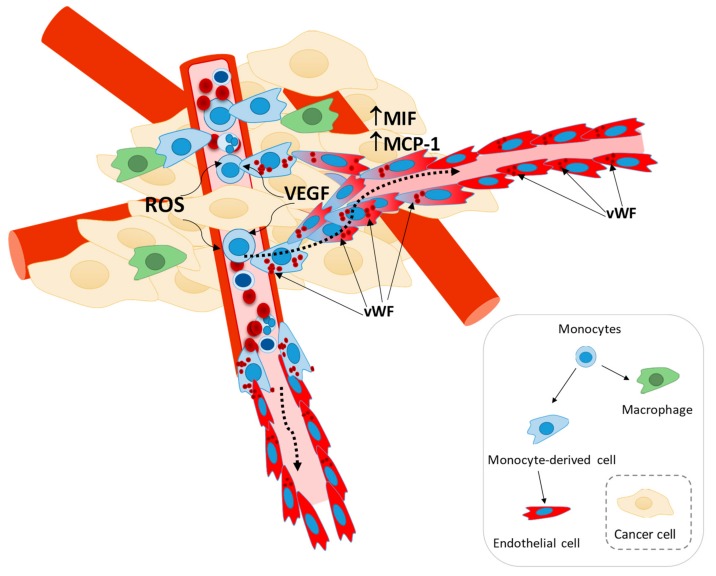
Monocytes recruitment into the tumor and endothelial differentiation. Our working model demonstrate that monocytes could act as EPCs and be incorporated into neo-vasculature. Monocytes are a cell subtype characterized as having high plasticity, and are able to differentiate into macrophages. We unravel that endothelial differentiation can also be an endpoint for monocytes. Monocyte–EC differentiation depends on a pro-angiogenic stimulus (e.g., VEGF) and oxidative stress (ROS). The acquisition of the expression of vWF (EC marker) is undoubtable evidence of this differentiation route, accounting for tumor vascularization and growth.

Our study demonstrated that monocytes are in fact incorporated in blood vessels, cementing their underestimation as a relevant stanchion of vascular growth. Due to their 2–10% prevalence in peripheral blood [50] and comparing to the estimated percentage of EPCs proposed by other studies [4,5,6,102,103], monocytes are putatively the most representative EPCs subgroup. Herein, we provided new evidence positioning a redox-dependence of monocytes under pro-angiogenic stimuli, which contributes for vascular growth (Figure 6). This new view on neoangiogenesis and ECs sources is worthy of further investigation, since a better understanding of monocytes biology as EPCs will propel novel paradigms for anti-angiogenic strategies and cancer therapy.

## Figures and Tables

**Table 1:** List of primers used for RQ-PCR.

Primer	Foward	Reverse
CXCR4	CTCCAAGCTGTCACACTCCA	TCGATGCTGATCCCAATGTA
CSF-1	GTCTTCCACCTGCTGGTGC	CCCTCTGGTTGCTCCAAGG
FLT1	CACCAAGAGCGACGTGTG	TTTTGGGTCTCTGTGCCAG
Hes1	ACGACACCGGATAAACCAAA	CGGAGGTGCTTCACTGTCAT
Ang1	GGGGAGGTTGGACTGTAATAC	GCATGTACTGCCTCTGACTG
Dll1	ATGCCTTCGGCCACTTCAC	CACATCCAGGCAGGCAGAT
Notch1	TGGCGGGAAGTGTGAAGCGG	GTGCTGAGGCACGGGTTGGG
Notch2	CCACAGGTGTCAGAATGGAG	GGCATTCATCCACATCCTCTG
Hey1	GACGAGACCGGATCAATAACAG	GGTCATCTGCAGGATCTCG
Hey2	GAGCGAGAACAATTACTCGGG	GTTATTTATCCGATCCCGACGC
FGFR4	GATGCTCAAAGACAACGCCTC	GACACCAAGCAGGTTGATGATG
18S	GCCCTATCAACTTTCGATGGT	CCGGAATCGAACCCTGATT

## Supplementary Materials

The following are available online at https://www.mdpi.com/2073-4409/9/1/107/s1, Figure S1: Representative histograms of FACS analysis of CD14—monocytic marker, CD31, KDR, VE-Cadherin (VE-Cad)—EC markers and CD68, CD80, CD163 – macrophage markers in monocytes freshly isolated (Day0), monocytes maintained in CFU media and in Human umbilical vein ECs (HUVECs), Figure S2: Confirmation of the specificity of anti- human vWF (anti-hvWF; A0082, Dako—Agilent) in lung section from mouse and human origin.
