# Supplementary material for: Monocytes as Endothelial Progenitor Cells (EPCs), Another Brick in the Wall to Disentangle Tumor Angiogenesis
Source: Cells. 2020 Jan 1;9(1):107. doi: 10.3390/cells9010107 (PMC7016533; doi:10.3390/cells9010107)
Supplement: Supplementary file 1 [file cells-09-00107-s001.pdf]

Supplementary Materials

Monocytes as Endothelial Precursor Cells (EPCs), Another Brick in the Wall to Disentangle Tumor Angiogenesis

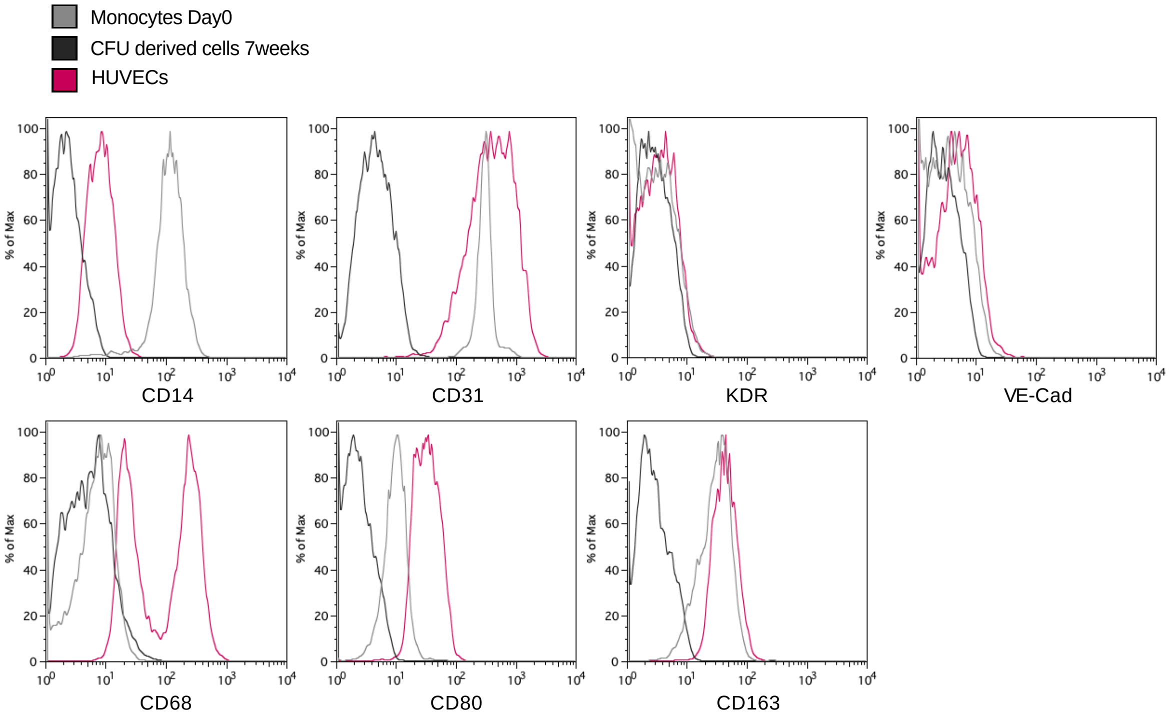

Figure S1. Representative histograms of FACS analysis of CD14 - monocytic marker, CD31, KDR, VE-Cadherin (VE-Cad) – EC markers and CD68, CD80, CD163 – macrophage markers in monocytes freshly isolated (Day0), monocytes maintained in CFU media and in Human umbilical vein ECs (HUVECs).

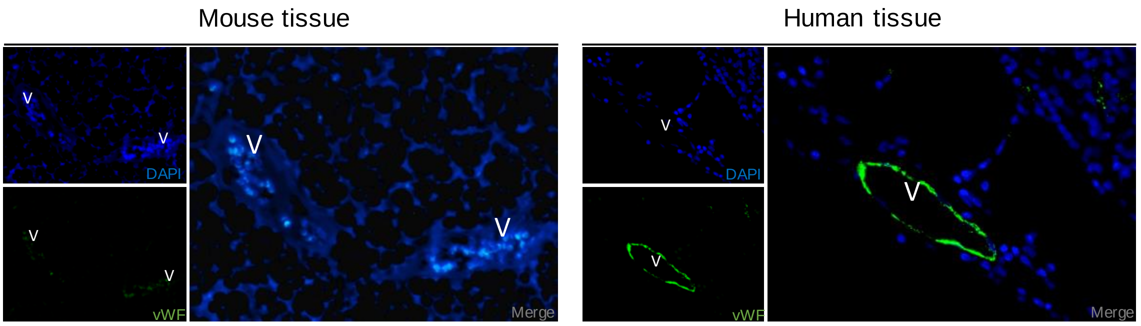

Figure S2. Confirmation of the specificity of anti- human vWF (anti-hvWF; A0082, Dako – Agilent) in lung section from mouse and human origin. Vessels (V) in mouse were negative for hvWF and human vessels were positive for hvWF (green) optimization in mouse and human tissues. Nuclei were counterstained with DAPI (blue).
